# Causal Effects of Gut Microbiota on Age-Related Macular Degeneration: A Mendelian Randomization Study

**DOI:** 10.1167/iovs.64.12.32

**Published:** 2023-09-19

**Authors:** Deshen Mao, Borui Tao, Shuyan Sheng, Hui Jin, Wenxuan Chen, Huimin Gao, Jianyi Deng, Zhuo Li, Fan Chen, Shixin Chan, Longqi Qian

**Affiliations:** 1 Department of Ophthalmology, Anqing Municipal Hospital, Anqing, China; 2 First Clinical Medical College, Anhui Medical University, Hefei, China; 3 Second Clinical Medical College, Anhui Medical University, Hefei, China; 4 Department of Medical Imaging, Anqing First People's Hospital, Anqing, China; 5 School of Clinical Medicine, Anhui Medical University, Hefei, China; 6 Department of Pathology, Anqing Municipal Hospital, Anqing, China; 7 Department of General Surgery, The First Affiliated Hospital of Anhui Medical University, Hefei, China

**Keywords:** mendelian randomization study, gut microbiota, age-related macular degeneration

## Abstract

**Purpose:**

Recently, the association between gut microbiota and age-related macular degeneration (AMD) through the gut–retina axis has attracted great interest. However, the causal relationship between them has not been elucidated. Using publicly available genome-wide association study summary statistics, we conducted a two-sample Mendelian randomization (MR) analysis to examine the causal relationship between the gut microbiota and the occurrence of AMD.

**Methods:**

The study used a variety of quality control techniques to select instrumental single nucleotide polymorphisms (SNPs) with strong exposure associations. We used a set of SNPs as instrumental variable that were below the genome-wide statistical significance threshold (5 × 10^−8^). Additionally, a separate group of SNPs below the locus-wide significance level (1 × 10^–5^) were selected as instrumental variables to ensure a comprehensive conclusion. Inverse variance-weighted (IVW) analysis was the primary technique we used to examine causality in order to confirm the validity of our findings. The MR-Egger intercept test, Cochran's *Q* test, and leave-one-out sensitivity analysis were used to evaluate the horizontal pleiotropy, heterogeneities, and stability of the genetic variants.

**Results:**

IVW results showed that genus *Anaerotruncus* (*P* = 5.00 × 10^−3^), genus *Candidatus Soleaferrea* (*P* = 1.83 × 10^−2^), and genus *unknown id.2071* (*P* = 3.12 × 10^−2^) were protective factors for AMD. The *Eubacterium oxidoreducens* group (*P* = 3.17 × 10^−2^), genus *Faecalibacterium* (*P* = 2.67 × 10^−2^), and genus *Ruminococcaceae UCG-011* (*P* = 4.04 × 10^−2^) were risk factors of AMD. No gut microbiota (GM) taxa were found to be causally related to AMD at the phylum, class, order, and family levels (*P* > 0.05). The robustness of MR results were confirmed by heterogeneity and pleiotropy analysis. (*P* > 0.05). We also performed a bidirectional analysis, which showed that genus *Anaerotruncus*, genus *Candidatus Soleaferrea*, genus *unknown id.2071* and the *Eubacterium oxidoreducens* group had an interaction with AMD, whereas genus *Faecalibacterium* showed only a unilateral unfavorable effect on AMD.

**Conclusions:**

We confirmed a causal relationship between AMD and GM taxa, including the *Eubacterium oxidoreducens* group, *Faecalibacterium*, *Ruminococcaceae UCG-011*, *Anaerotruncus*, and *Candidatus Soleaferrea*. These strains have the potential to serve as new biomarkers, offering valuable insights into the treatment and prevention of AMD.

Age-related macular degeneration (AMD) is one of the diseases leading to blindness worldwide and affecting millions of people.^1^ There are two types of AMD: neovascular AMD and non-neovascular AMD, which also can be called wet AMD and dry AMD, respectively. Dry AMD is the most common type and can progress to wet AMD. Wet AMD is more severe and accounts for approximately 80% of vision loss as a result of AMD due to the hemorrhaging and exudation in the retina.^2^^,^^3^ AMD causes almost 9% of all cases of blindness worldwide, and the number of people with AMD is stably increasing, projected to be 300 million by 2040.^4^^,^^5^ Several risk factors have been identified to contribute to the complex mechanism of AMD, including age, smoking, increased body mass index, hypertension, hyperlipidemia, and genetics. Among these, age is regarded as the most prominent risk factor.^4^^,^^6^ The identification of these mechanisms has allowed researchers to develop more precise treatment and prevention methods for AMD. Nevertheless, the exact pathophysiology of the disease still remains unclear.

The gut microbiota (GM) has been found to have a strong connection with the development of inflammatory, metabolic, mental, and immune diseases, as well as neurotransmitter function.^7^^–^^11^ Recent research has shown that there is a link between the GM and AMD through what is known as the gut–retina axis.^5^ The findings of Rowan and Taylor^12^ suggest that a high glycemic index diet is linked to specific histological features of dry AMD. Zinkernagel et al.^13^ reported that patients with AMD had elevated levels of *Streptococcus* and *Gemella* species and decreased levels of *Prevotella* and *Leptotrichia* species in comparison to the control group.

The relationship between the GM and AMD is complex, with numerous factors such as diet and rest affecting GM composition. Although randomized controlled trials (RCTs) could provide definitive evidence of a causal relationship between the GM and AMD, they are limited by practical and ethical concerns. Conducting RCTs is a time-consuming and resource-intensive process, making it difficult to conduct large-scale studies in this area. Consequently, research on the link between the GM and AMD remains insufficient.

Mendelian randomization (MR) is a research method that uses genetic variation to assess the causal effects of functions or phenotypes on disease outcomes, similar to the design of RCTs. However, instead of using different treatments, MR uses instrumental variables (IVs) to control for potential confounding factors. If there exists an instrumental variable that is linked to the exposure but has no association with any confounding factor influencing the relationship between the exposure and the outcome, and if there is no direct causal connection from the instrumental variable to the outcome except through the exposure, it is possible to estimate the causal effect of the exposure on the outcome assuming either a single instrumental variable or that a set of instrumental variables for the exposure is accessible. MR has been widely used in various fields and has yielded important findings. To explore the causal relationship between the GM and AMD, we chose GM taxa as the exposure and AMD as the outcome for MR analysis.

## Materials and Methods

### Assumptions and Study Design of MR

**Figure 1.**
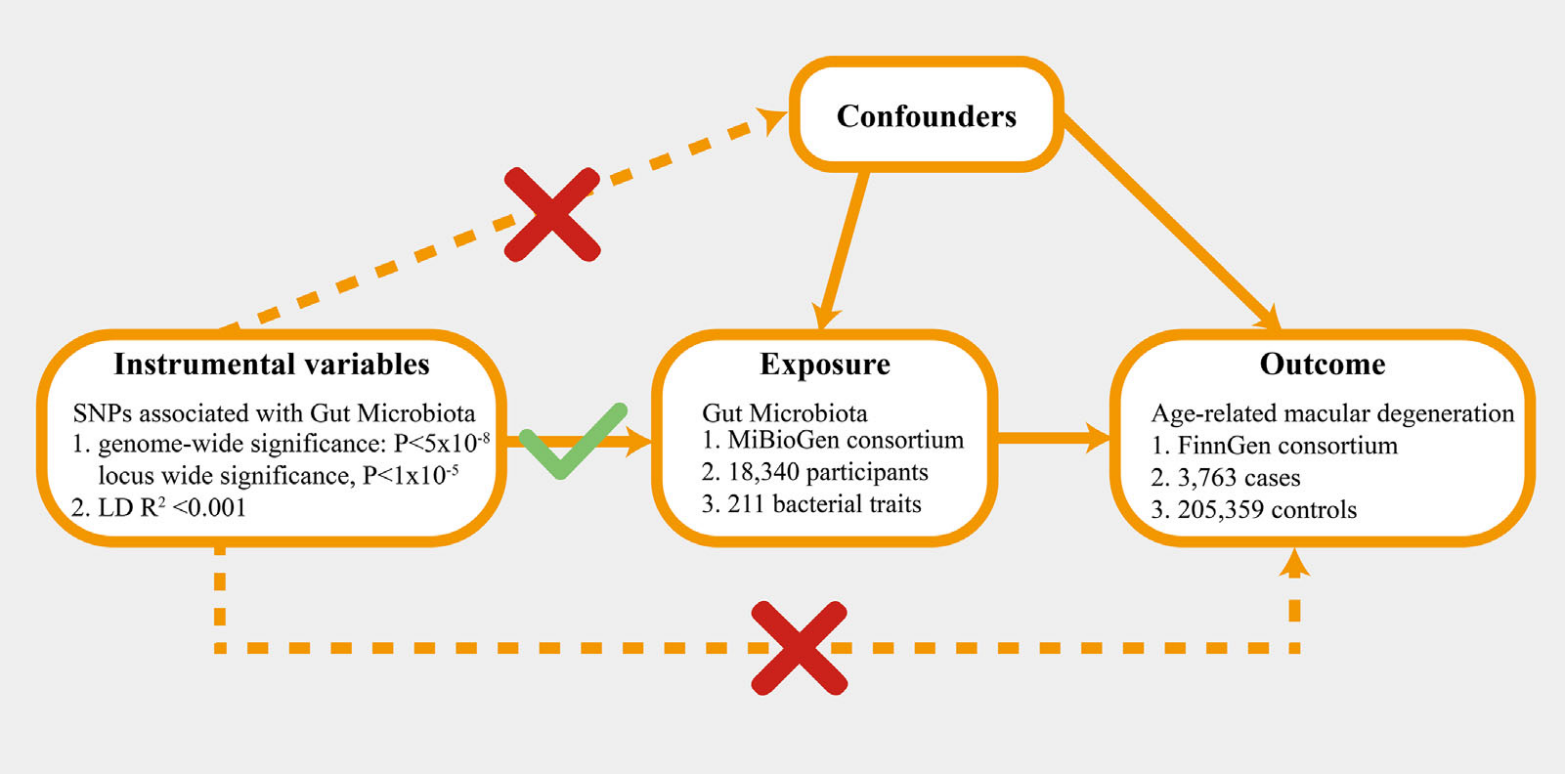
Overview of MR analyses process and major assumptions.

In this study, we conducted a two-sample MR analysis to evaluate the causal relationship between GM taxa and AMD, utilizing publicly accessible summary-level data from genome-wide association studies (GWASs) for both the exposures (GM taxa) and the outcome (AMD). To guarantee the validity of the MR analysis, three assumptions had to be met: (1) The genetic variants used in the analysis should have a significant association with the exposure; (2) the genetic variants selected as IVs for exposure should be uncorrelated with confounding factors that are linked to both the exposure and outcome; and (3) there should be no horizontal pleiotropy, meaning that IVs can only affect AMD through GM taxa (Fig. 1).^14^

### Ethics Statement

This study made use of deidentified public summary-level data, which can be downloaded for free, to analyze the relationship between GM taxa and AMD. The GWASs used in this investigation were all approved by their respective institutional ethics committees.

### Exposure Sources of GM Taxa

Kurilshikov and colleagues^15^ used data from the MiBioGen consortium to investigate the link between the GM and genetic variation. The dataset consisted of 16S rRNA gene sequencing profiles and genotyping information from 18,340 individuals of European ancestry, recruited from 25 cohorts in 11 countries. Using this information, the team identified 122,110 variant sites across 211 taxa (from genus to phylum level) to analyze the variation in GM taxa across diverse populations. Among the 211 taxa, we deleted four of them because three of them showed no result in the heterogeneity and pleiotropy analysis, and the *P* value of one of them was out of 1 × 10^−5^. From the MiBioGen consortium GWAS, we identified IVs representing GM taxa at five taxonomic levels. Further information on the GM data utilized in this study can be accessed in the primary publication.^15^^,^^16^

In order to meet the three basic assumptions of MR and ensure the accuracy of the results, we performed quality checks on all of the single nucleotide polymorphisms (SNPs). To guarantee that the SNPs we selected were significantly associated with the exposure, all SNPs associated with GM taxa reached the genome-wide significance threshold (*P* < 5 × 10^−8^). Additionally, a separate group of SNPs below the locus-wide significance level (1 × 10^−5^) were selected as instrumental variables to ensure a comprehensive conclusion. Linkage disequilibrium (LD) analysis (*R*^2^ < 0.001, clumping distance = 10,000 kb) was also performed to meet the MR assumptions. In order to prevent the influence of alleles on results for the causal relationship between GM taxa and AMD, palindrome SNPs were removed.

In order to mitigate the risk of potential weak instrumental bias, the strength of the IVs was evaluated using the *F* statistic, which was calculated by the following formula: *F* =  *R*^2^ × (N – 2)/(1 – R^2^), where *N* refers to the sample size. A correlation between an IV and exposure was deemed sufficiently robust to safeguard the results of the MR analysis against weak instrumental bias if the *F* statistic exceeded 10.^17^

### Outcome Source of AMD

The summary-level data for AMD were extracted from a large-scale mate-analysis GWAS including 3763 cases and 205,359 controls and 16,380,424 variables from the FinnGen biobank analysis round 5.

### Statistical Analysis

R 4.1.1 (R Foundation for Statistical Computing, Vienna, Austria) was utilized to conduct all statistical analyses in this study. The TwoSampleMR package in R was used to perform the MR analysis to investigate the potential causal relationship between GM taxa and AMD. A statistical significance level of *P* < 0.05 was adopted to indicate evidence of a potential causal effect.

#### MR Estimates

We used a variety of techniques, such as inverse variance-weighted (IVW) analysis, the weighted mean, the weighted median (WM), and the MR-Egger test, to confirm the validity of the study. To obtain overall estimates of the impact of the GM on AMD,^18^ the IVW analysis technique combined Wald estimates for each SNP using a meta-analysis methodology. We selected the fixed or random effects model for the IVW test depending on the presence or absence of heterogeneity. In situations where considerable heterogeneity (*P* < 0.05) was identified, we employed a random-effect IVW model. As supplemental analyses, we also ran the WM technique and MR-Egger test. If the proportion of SNPs with heterogeneity was greater than 50%, we regarded the WM data as indicating strong causal effects. The results of MR-Egger were deemed reliable if the proportion of pleiotropic SNPs was more than 50%. Nonetheless, it is worth mentioning that MR-Egger estimations may be erroneous and highly impacted by outlying genetic variations. All statistical analyses were done using R 4.1.1, and *P* < 0.05 was regarded as the threshold for statistical significance.^14^

#### Sensitivity Analysis

In this study, we employed the MR-Egger as well as MR-PRESSO regression methods to evaluate the potential presence of pleiotropy in the SNPs used as IVs. We considered horizontal pleiotropy to be absent if *P* > 0.05. Heterogeneity was evaluated using Cochrane's *Q* test, and IVs with *P* < 0.05 were deemed heterogeneous. Furthermore, we carried out a sensitivity analysis known as leave one out, in which each SNP was excluded in turn during the MR analysis to identify any potentially influential SNPs.

## Results

### Selection of IVs Related to the GM

Following quality control measures including LD effects and palindromic analysis, a total of 2282 SNPs were found to be IVs associated with 211 bacterial taxa for AMD (with a significance threshold of *P* < 1 × 10^−5^). These IVs were found in a diverse set of taxa, including nine phyla (with 106 SNPs), 16 classes (with 185 SNPs), 20 orders (with 227 SNPs), 35 families (with 388 SNPs), and 131 genera (with 1376 SNPs). Notably, each SNP demonstrated adequate validity, with values ranging from 16.91 to 88.43, and all *F* values were greater than 10. Supplementary Table S1 details the major information.

Only 16 SNPs passed quality control measures and were found suitable to be utilized as IVs when considering the GM as a whole (with a significance threshold of *P* < 5 × 10^−8^). Notably, each of these SNPs demonstrated adequate validity, with values ranging from 29.81 to 88.43 and all *F* values greater than 10. Additionally, 25 SNPs were identified as IVs that were associated with 211 bacterial taxa for AMD (with a significance threshold of *P* < 5 × 10^−8^). Supplementary Table S2 details the major information. These IVs were distributed among a total of 13 genera (with 14 SNPs), five families (with six SNPs), two orders (with three SNPs), one class (with one SNP), and one phylum (with one SNP). Detailed information regarding the IVs is provided in Table 1.

**Table 1.** Selection of IVs After Quality Control

Taxonomies	Taxa, *n*	IVs, *n*
Phylum	9	106
Class	16	185
Order	20	227
Family	35	388
Genus	131	1376
Total	211	2282

### Results of MR Analysis

**Figure 2.**
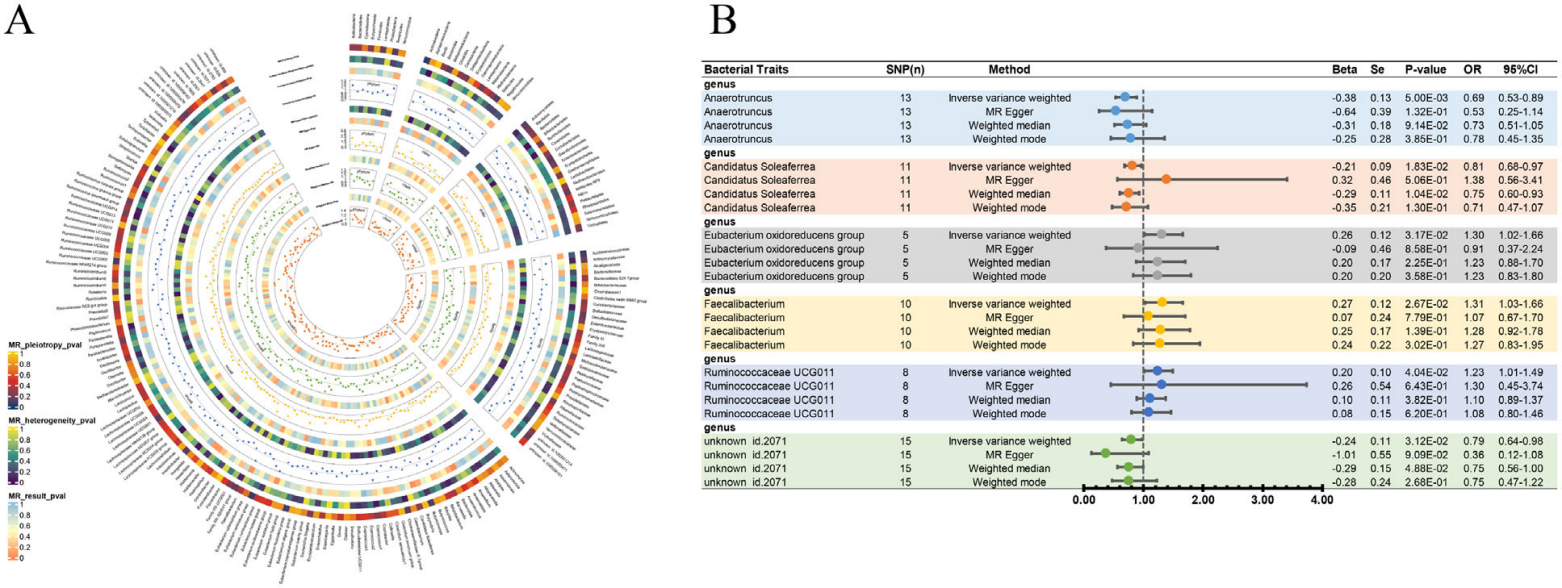
Causal analysis of GM and AMD. (**A**) All results of MR analysis and sensitivity analysis between GM and AMD. (**B**) MR results of GM taxa with a causal relationship to AMD.

As part of the MR analysis, we observed a genetically predicted relative abundance of six different genera. Furthermore, Figure 2A provides a visual representation of the relationship between 211 bacterial taxa and AMD. The MR estimates of IVW indicated that the *Eubacterium oxidoreducens* group (beta [β] = 0.26; standard error [SE] = 0.12; odds ratio [OR] = 1.30; 95% confidence interval [CI], 1.02–1.66; *P* = 3.17 × 10^−2^), *Faecalibacterium* (β = 0.27; SE = 0.12; OR = 1.31; 95% CI, 1.03–1.66; *P* = 2.67 × 10^−2^), and *Ruminococcaceae UCG-011* (β = 0.20; SE = 0.10; OR = 1.23; 95% CI, 1.01–1.49; *P* = 4.04 × 10^−2^) were risk factors for AMD. *Anaerotruncus* (β = −0.38; SE = 0.13; OR = 0.69; 95% CI, 0.53–0.89; *P* = 5.00 × 10^−3^), *Candidatus Soleaferrea* (β = −0.21; SE = 0.09; OR = 0.81; 95% CI, 0.68–0.97; *P* = 1.83 × 10^−3^), and *unknown id.2071* (β = −0.24; SE = 0.11; OR = 0.79; 95% CI, 0.64–0.98; *P* = 3.12 × 10^−2^) were considered to provide a protective effect (Fig. 2B). Other results are detailed in Supplementary Table S3. When the GM was considered as a whole, it showed a protective effect of AMD although it was not significant (IVW: β = −0.04; SE = 0.07; OR = 0.96, 95% CI, 0.83–1.11; MR-Egger: β = −0.26; SE = 0.24; OR = 0.77; 95% CI, 0.48–1.25; WM: β = −0.13; SE = 0.10; OR = 0.88; 95% CI, 0.72–1.07). Due to the small number of IVs that satisfied the criterion, none of the MR findings for individual classifications of bacterial taxa at 5 levels demonstrated a significant causal connection with AMD *(P* > 0.05) Supplementary Table S4 details the major information. In order to investigate the association of genetically predicted AMD on the GM, we performed a bidirectional analysis on GM-related SNPs (rs4980260, rs10754199, rs11200630, rs429608, and rs7478014). The results showed that genus *Anaerotruncus*, genus *Candidatus Soleaferrea*, genus *unknown id.2071*, and the *Eubacterium oxidoreducens* group had an interaction with AMD, whereas genus *Faecalibacterium* only showed a unilateral unfavorable effect on AMD (Table 2).

**Table 2.** Reverse MR Results Between AMD and GM (*P* < 5 × 10^−8^)

Exposure	Outcome	Method	nsnp	Beta	SE	Pval	OR	OR_lci95	OR_uci95
AMD	*Anaerotruncus*	Inverse variance weighted	5	0.025	0.012	0.032	1.025	1.002	1.049
AMD	*Anaerotruncus*	MR-Egger	5	0.007	0.024	0.778	1.007	0.962	1.055
AMD	*Anaerotruncus*	Weighted median	5	0.022	0.013	0.092	1.022	0.996	1.048
AMD	*Anaerotruncus*	Weighted mode	5	0.023	0.013	0.166	1.023	0.996	1.051
AMD	*Candidatus Soleaferrea*	Inverse variance weighted	5	0.010	0.019	0.620	1.010	0.972	1.048
AMD	*Candidatus Soleaferrea*	MR-Egger	5	0.026	0.039	0.545	1.027	0.951	1.108
AMD	*Candidatus Soleaferrea*	Weighted median	5	0.016	0.021	0.453	1.016	0.975	1.059
AMD	*Candidatus Soleaferrea*	Weighted mode	5	0.016	0.022	0.494	1.016	0.974	1.061
AMD	*Eubacterium oxidoreducens* group	Inverse variance weighted	5	0.021	0.021	0.307	1.021	0.981	1.063
AMD	*Eubacterium oxidoreducens* group	MR-Egger	5	0.037	0.041	0.433	1.038	0.957	1.126
AMD	*Eubacterium oxidoreducens* group	Weighted median	5	0.022	0.022	0.326	1.022	0.978	1.068
AMD	*Eubacterium oxidoreducens* group	Weighted mode	5	0.023	0.023	0.372	1.023	0.978	1.070
AMD	*Faecalibacterium*	Inverse variance weighted	5	−0.007	0.011	0.538	0.993	0.971	1.015
AMD	*Faecalibacterium*	MR-Egger	5	0.001	0.023	0.964	1.001	0.958	1.047
AMD	*Faecalibacterium*	Weighted median	5	−0.004	0.012	0.742	0.996	0.972	1.020
AMD	*Faecalibacterium*	Weighted mode	5	−0.004	0.013	0.795	0.996	0.971	1.022
AMD	*Ruminococcaceae UCG* *-* *011*	Inverse variance weighted	5	0.019	0.025	0.456	1.019	0.970	1.070
AMD	*Ruminococcaceae UCG* *-* *012*	MR-Egger	5	−0.025	0.050	0.657	0.976	0.884	1.077
AMD	*Ruminococcaceae UCG* *-* *013*	Weighted median	5	0.013	0.030	0.667	1.013	0.955	1.075
AMD	*Ruminococcaceae UCG* *-* *014*	Weighted mode	5	0.003	0.030	0.934	1.003	0.945	1.063
AMD	*U* *nknown genus id.2071*	Inverse variance weighted	5	0.015	0.013	0.260	1.015	0.989	1.042
AMD	*Unknown* *genus id.2071*	MR-Egger	5	0.028	0.027	0.373	1.029	0.975	1.085
AMD	*U* *nknown genus id.2071*	Weighted median	5	0.020	0.015	0.198	1.020	0.990	1.051
AMD	*U* *nknown genus id.2071*	Weighted mode	5	0.022	0.016	0.244	1.023	0.990	1.056

nsnp, Number of SNP.

### Sensitivity Analysis

In sensitivity analysis, in order to ensure the accuracy of our results we confirmed the effects of accurate MR results in six genera on AMD. No horizontal pleiotropy was observed in *Anaerotruncus* (*P* = 0.49; MR-PRESSO *P* = 0.75), *Candidatus Soleaferrea* (*P* = 0.27; MR-PRESSO *P* = 0.22), *Eubacterium oxidoreducens group* (*P* = 0.48; MR-PRESSO *P* = 0.25), *Faecalibacterium* (*P* = 0.35; MR-PRESSO *P* = 0.90), Ruminococcaceae *UCG-011* (P = 0.92; MR-PRESSO *P* = 0.15), and *unknown id.2071* (*P* = 0.18) for AMD. At the same time, no heterogeneity was found in *Anaerotruncus* (IVW *P* = 0.69; MR-Egger *P* = 0.65), *Candidatus Soleaferrea* (IVW *P* = 0.68; MR-Egger *P* = 0.73), *Eubacterium oxidoreducens group* (IVW *P* = 0.66; MR-Egger *P* = 0.62), *Faecalibacterium* (IVW *P* = 0.85; MR-Egger *P* = 0.87), Ruminococcaceae *UCG-011* (IVW *P* = 0.11; MR-Egger *P* = 0.07), and *unknown id.2071* (IVW *P* = 0.56; MR-Egger *P* = 0.65) for AMD (Table 3). Viewing the GM as a whole, the sensitivity analysis demonstrated no horizontal pleiotropy (*P* = 0.37; MR-PRESSO *P* = 0.13) or heterogeneity (IVW *P* = 0.31; MR-Egger *P* = 0.31) (Table 4). Supplementary Table S5 shows the pleiotropy and heterogeneity test results for all bacterial taxa and the GM viewed as a whole. Meanwhile, the leave-one-out results further validated data robustness (Supplementary Figs. S1–S6). In the absence of heterogeneity and pleiotropy, the results of IVW were trustworthy. Therefore, *Anaerotruncus*, *Candidatus Soleaferrea*, *Eubacterium oxidoreducens group*, *Faecalibacterium*, Ruminococcaceae *UCG-011*, and *unknown id.2071* were causally related to AMD*.*

**Table 3.** Sensitivity Analysis Between GM and AMD

Genus	Method	*Q*	*P*	Intercept	*P*
*Eubacterium oxidoreducens* group	IVW	2.44	0.66	0.04	0.48
	MR-Egger	1.8	0.62	—	—
*Faecalibacterium*	IVW	4.86	0.85	0.02	0.35
	MR-Egger	3.88	0.87	—	—
*Ruminococcaceae* *UCG-011*	IVW	11.59	0.11	−73	0.92
	MR-Egger	11.57	0.07	—	—
*Anaerotruncus*	IVW	9.2	0.69	0.02	0.49
	MR-Egger	8.7	0.65	—	—
*Candidatus Soleaferrea*	IVW	7.46	0.68	−0.05	0.27
	MR-Egger	6.1	0.73	—	—
*Unknown* *id.2071*	IVW	12.53	0.56	0.06	0.18
	MR-Egger	10.49	0.65	—	—

**Table 4.** MR Results Between GM and AMD (*P* < 5 × 10^−8^)

GM	Method	IVs	OR	95%CI	*P*	*Q*	*Q*-*P*	Intercept	*P*
Total	Inverse variance weighted	16	0.96	0.83–1.11	0.57	17.09	0.31	2.53 × 10^−2^	0.37
Total	Weighted mode	16	0.85	0.67–1.08	0.20	—	—	—	—
Total	Weighted median	16	0.88	0.72–1.07	0.21	—	—	—	—
Total	MR-Egger	16	0.77	0.48–1.25	0.31	16.11	0.31	—	—

## Discussion

To mitigate the potential confounding effects of factors such as diet and rest, we employed MR analysis to evaluate the possible causal link between GM taxa and AMD. Our analyses did not uncover any significant associations between GM taxa and AMD risk at the phylum, class, order, or family levels. However, we did identify a total of three different genus taxa that were correlated with a decreased risk of AMD, as well as three other genus taxa that were associated with an increased risk of AMD. These findings have important implications for the identification of novel biomarkers in future AMD investigations and may inspire novel prevention and therapeutic strategies for this condition.

The GM is composed of various microorganisms, including bacteria, viruses, fungi, and archaea, that reside in the human digestive tract. These microorganisms play a critical role in various physiological and metabolic functions, including the digestion and absorption of nutrients, development of the immune system, and production of essential vitamins.^19^ The composition of the GM varies depending on various factors such as age, diet, lifestyle, and geographical location.^20^ However, there are some predominant bacterial species that are commonly found in the gut microbiota of healthy individuals. These include Bacteroidetes, Firmicutes, Actinobacteria, Proteobacteria, and Verrucomicrobia*.*^21^ Dysbiosis of the GM can cause various diseases in the body, including intestinal diseases, metabolic diseases, autoimmune diseases, neurological diseases, and so on.^22^^–^^25^

Recently, the gut–retina axis has attracted attention. The retina is regarded as a unique tissue in terms of its immune response, as it benefits from multiple protective layers, including the inner and outer blood–retina barrier and the blood–aqueous barrier. In addition to these physical barriers, the retina also employs resistance and tolerance mechanisms to defend against any potential threats from both internal and external sources. Moreover, the retina has its own intrinsic defense mechanisms, such as microglia and the complement system, which help to maintain its normal function and protect against damage.^26^^–^^28^ Increased intestinal permeability is associated with gut dysbiosis, which impairs the metabolism and absorption of macro- and micronutrients in the gut barrier.^29^ The second mechanism allows for enhanced mobility of bacterial compounds, including the endotoxin lipopolysaccharides and pathogen-associated molecular pattern (PAMP) molecules. This can trigger low-level inflammation in various tissues by activating pattern-recognition receptors (PRRs).^5^ When these biological processes occur in retina, different types of PRRs that can be stimulated by PAMPs originating from the intestines are expressed by microglia, perivascular macrophages, certain dendritic cells, and RPE cells. This activation can contribute to inflammation in the eye.^30^ When considered collectively, the data clearly point to the presence of a gut–retina axis that is important for the onset and development of ocular disorders. This viewpoint is also supported by the pertinent studies. AMD, diabetic retinopathy, glaucoma, and other retinal neurodegenerative disorders can all be brought on by dysbiosis.^31^

AMD is a complex disorder whose pathogenesis is influenced by a number of interrelated factors, including aging-related changes and a confluence of environmental, epigenetic, and genetic factors. Currently, lifestyle, diet, the immune system (immunosenescence), and sterile low-grade chronic inflammation (inflammaging) are considered to be main causes.^32^ The risk factors for AMD significantly overlap with molecular processes generated by GM dysbiosis. Because of this, AMD is thought to be most closely related to ophthalmic conditions brought on by GM disorders. Early life's microbiome is very changeable and affected by factors including delivery method, diet, family environment, geographic location, genetics, and antibiotic use. As a child gets closer to becoming an adult, the microbiome becomes more stable. In older age, alterations in the microbiome are connected with degenerative disorders, including an altered Bacteroidetes-to-Firmicutes ratio,^33^ which has been linked to AMD.^13^ These modifications in the composition of the microbiome have the potential to either influence host metabolism or exert stress on it, thereby serving as potential contributors to inflammation and disease.^34^ Studies also have shown that the intestinal microbiome may trigger autoimmune responses in the eye through activation signals to retina-specific T cells,^35^ and the GM also seems to be associated with the complement system in the occurrence of AMD.^36^ Age-related gut dysbiosis has been linked to increased intestinal permeability; persistent, low-grade inflammation; and elevated levels of proinflammatory cytokines and vascular endothelial growth factor, as previously mentioned. Ultimately, these processes may contribute to the pathological angiogenesis that is often observed in AMD.^37^ On the other hand, given the link between AMD and diet, the composition of the intestinal microbiome may also influence AMD development and progression, as diet has been proved to be a risk factor for AMD, and the GM play an important role in the digestion of food and influence the body's global metabolism.^38^^,^^39^ The GM can also influence the pathological angiogenesis in AMD. Studies have shown that a high-fat diet modulates the GM and exacerbates choroidal neovascularization (CNV). Another study also confirmed that a high-fat diet aggravates CNV through the GM.^37^ In conclusion, identifying the composition of the GM to detect specific diagnostic markers for AMD is considered worthy of further research.^40^ Despite some pioneering work, research on the gut–retina axis is still in its infancy. Through MR analysis, we propose new possible research directions.

Zinkernagel et al.^13^ noted a significant change in the relative abundance of Firmicutes and Bacteroidetes at the phylum level among AMD patients, with a relative increase in Firmicutes and a decrease in Bacteroidetes. This observation is consistent with previous studies indicating that a high ratio of Firmicutes to Bacteroidetes is often associated with obesity, which is itself a risk factor for AMD.^41^ This is consistent with our research. Here, we observed that the *Eubacterium oxidoreducens* group, *Faecalibacterium*, and Ruminococcaceae *UCG-011* are risk factors for AMD. It is worth noting that all of them are Firmicutes. In a study using a mouse model, it was demonstrated that high-fat diets can worsen choroidal neovascularization by increasing the prevalence of Firmicutes bacteria. These observations were linked to an increase in intestinal permeability and chronic inflammation, with elevated levels of interleukin (IL)-6 and IL-1b, tumor necrosis factor-alpha (TNF-α), and vascular endothelial growth factor A (VEGF-A) cytokines. These cytokines have been previously associated with the progression of neovascular AMD.^37^ From the phylum standpoint, the effect of Firmicutes increases the risk of the onset of AMD perhaps both directly and indirectly, because, with the increase of Firmicutes*,* there is often a decrease in Bacteroides. The generation of volatile fatty acids from the fermentation of carbohydrates, which is then reabsorbed through the intestinal mucosa and serves as a key source of energy for the host, is known to be significantly influenced by the Bacteroides genera*.*^42^ Moreover, Bacteroides has the capacity to produce polysaccharide A (PSA), which, through interactions with ligand receptors, may be implicated in the control of the immune response to pathogens. In fact, research has demonstrated that PSA produced from Bacteroides species can guard against autoimmune encephalitis in an experimental setting.^43^ A more detailed analysis of the microbiota in patients with AMD revealed significant increases in the relative abundances of two specific bacterial taxa: *Ruminococcus torques*, a Gram-positive bacterium known for its ability to degrade mucin, and *Oscillibacter*, which has been previously linked to high-fat diets.^44^ These findings suggest that these particular bacteria may play a role in the development or progression of AMD. This is consistent with our analysis results, which indeed suggest that they are risk factors for AMD. Recent studies have suggested that specific bacterial taxa, including *Oscillibacter*, *Anaerotruncus*, and *Eubacterium ventriosum* spp., may contribute to the pathogenesis of age-related diseases such as AMD. Increased populations of *Oscillibacter* have been associated with heightened gut permeability, potentially due to a reduction in the mRNA expression of tight junctions such as zonula occludens 1 (ZO-1).^45^ Likewise, elevated levels of *Anaerotruncus* species have been linked to aging and age-associated inflammation in a mouse model, with corresponding increases in proinflammatory chemokines.^46^ In humans, high levels of *Eubacterium ventriosum* spp. have been associated with elevated levels of proinflammatory cytokines such as IL-6 and IL-8.^47^ These findings provide important insights into the mechanisms underlying the development and progression of AMD. Overall, our predictions are consistent with the data previously obtained in the laboratory, and the relevant mechanisms have also been reported. It is logical that the *Eubacterium oxidoreducens* group, *Faecalibacterium*, and *Ruminococcaceae UCG-011*, as risk factors for AMD, have shown increased abundance in AMD compared to control groups. However, the research by Zinkernagel et al.^13^ about *Anaerotruncus* is not consistent with the results of our study. They pointed out this bacterium increased when triggered by inflammation. However, in the overall context of the disease, we believe that it has a protective effect against AMD. This may be because there are not many SNPs acting as instrumental factors, and they only account for causation in a limited way. However, this cannot completely negate the possibility of *Anaerotruncus* being protective. Inflammation is a double-edged sword in the onset of diseases. The abnormal proliferation of *Anaerotruncus* may be due to the overall growth suppression of other phyla by Firmicutes, creating favorable conditions for the proliferation of *Anaerotruncus*. In summary, we have raised an intriguing question, and further laboratory validation is necessary. Let us focus on another protective role on AMD that we observed. The hallmark of neovascular or wet AMD, one of the advanced stages of AMD, is the presence of CNV.^48^ Li et al.^49^ found that, compared to normal mice, the abundance of *Candidatus Soleaferrea* was significantly downregulated. In CNV mice, the proportion of *Candidatus Saccharimonas* increased and the proportion of *Candidatus Soleaferrea* decreased. This suggests that, perhaps in normal mice, *Candidatus Soleaferrea* may play a protective role by resisting the metabolic pathway changes and inflammation induced by *Candidatus Saccharimonas*. Hence, although the precise mechanisms remain unclear, there is a possibility that *Candidatus Saccharimonas* is linked to inflammation and the resulting immune response of the host.^50^

This study has several notable strengths. First, unlike previous investigations on the association between GM and ocular disorders that have mostly concentrated on family-level classification, our analysis took a more granular approach by examining the causal impact of each GM taxon on AMD from the genus to the phylum level. This approach provided a conceptual framework for probing the mechanisms of particular bacterial strains on AMD and offered a wealth of valuable clinical insights, such as that the increase of *Ruminococcaceae* and decrease in *Candidatus Soleaferrea* could be considered to be related to a high-fat diet. This may be a targeted treatment approach to reducing the incidence of AMD caused by a high-fat diet.^51^ Second, the utilization of the latest large-scale GWASs allowed for the analysis of genetic data from a substantial sample size, lending greater credibility to our findings when compared to smaller randomized controlled studies. Additionally, the use of MR analysis helps avoided confusion and provided a fresh perspective for exploring the mechanisms of the gut–retina axis.

Although this study complied with the presumptions of MR analyses, there are several restrictions that should be taken into account. If the IVs employed are tightly linked with GM taxa, there is still a chance of minor instrumental bias, as with previous MR studies that have concentrated on GM. Furthermore, the cohort used for the AMD analysis from the FinnGen program resulted in several limitations in our study, including that quality checking to ensure the accuracy of diagnoses was missing, and there are not sufficient control of age or any other factors among the patients included in the study. In the future, building upon this study, we will conduct further research. We plan to utilize data from multiple centers. We will replicate our findings with the AMD cohort from the large International AMD Genetics consortium and combine it with information from our own collected cases to make this study more comprehensive. Moreover, applying several statistical adjustments could be unduly strict and cautious, resulting in missing GM taxa that might have a possible causal connection to AMD. Hence, in light of the biological plausibility of our findings, we did not examine the results of repeated testing. Despite being the first study to use MR analysis to examine the relationship between GM taxa and AMD risk at the species level, to the best of our knowledge, we were unable to establish a causal relationship between AMD and any particular GM species. To provide additional theoretical evidence for the examination of the gut–retina axis mechanism, future studies with a bigger sample size are required to look into the connection between GM taxa and AMD at the species level.

In conclusion, our work demonstrated a causal relationship between the risk of AMD development and particular GM taxa, such as the *Faecalibacterium*, *Ruminococcaceae* *UCG-011*, *Anaerotruncus*, and *Candidatus Soleaferrea*. Our results suggest that these GM taxa may provide new opportunities for the development of AMD treatments and preventative measures, as well as potential biomarkers.

## Supplementary Material

Supplement 1

Supplement 2
