# Supplementary material for: Causal Effects of Gut Microbiota on Age-Related Macular Degeneration: A Mendelian Randomization Study
Source: Invest Ophthalmol Vis Sci. 2023 Sep 19;64(12):32. doi: 10.1167/iovs.64.12.32 (PMC10513115; doi:10.1167/iovs.64.12.32)
Supplement: Supplement 1 [file iovs-64-12-32_s001.pdf]

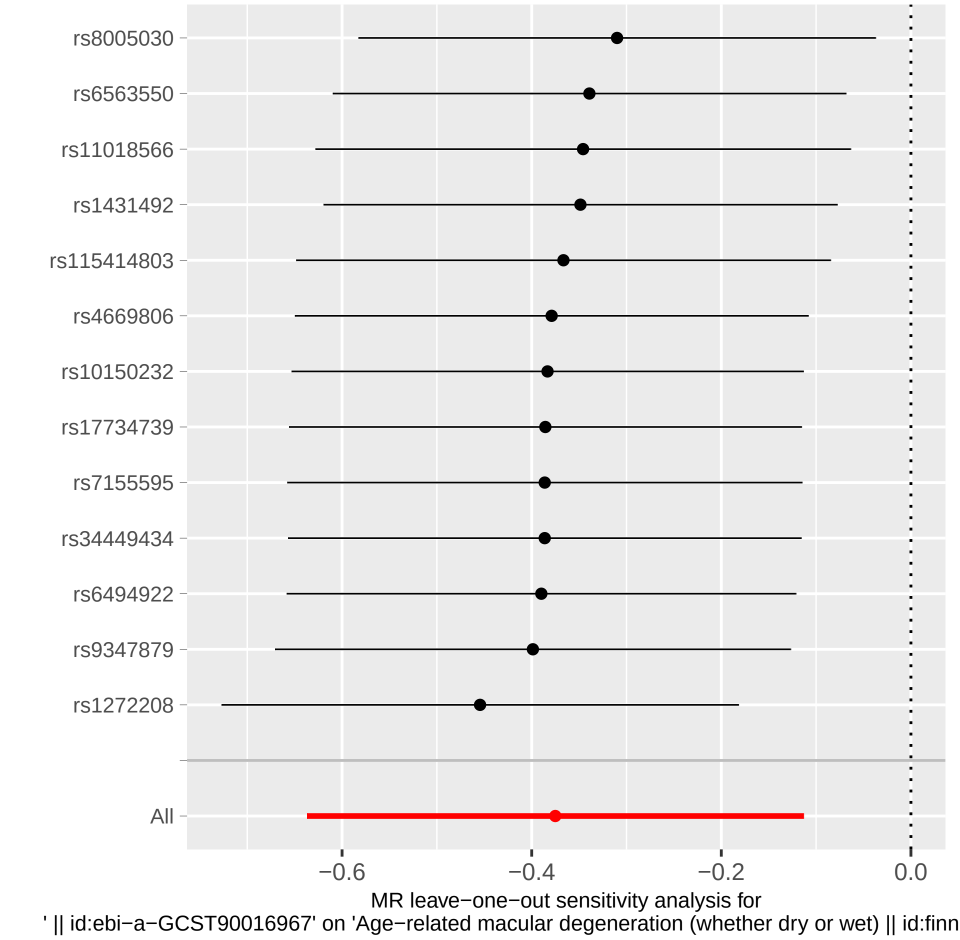
rs8005030
rs6563550
rs11018566
rs1431492
rs115414803
rs4669806
rs10150232
rs17734739
rs7155595
rs34449434
rs6494922
rs9347879
rs1272208
All
−0.6
−0.4
−0.2
0.0
MR leave−one−out sensitivity analysis for
' || id:ebi−a−GCST90016967' on 'Age−related macular degeneration (whether dry or wet) || id:finn

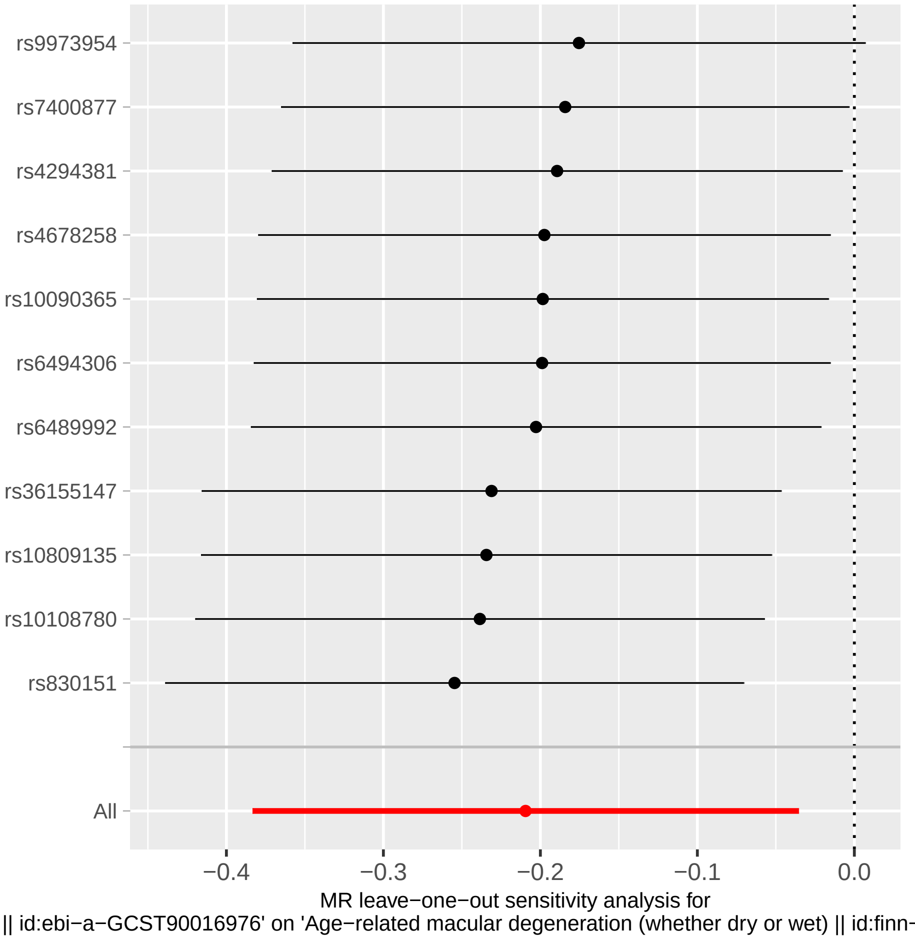

rs9973954
rs7400877
rs4294381
rs4678258
rs10090365
rs6494306
rs6489992
rs36155147
rs10809135
rs10108780
rs830151
All
−0.4
−0.3
−0.2
−0.1
0.0
MR leave−one−out sensitivity analysis for
' || id:ebi−a−GCST90016976' on 'Age−related macular degeneration (whether dry or wet) || id:finn−

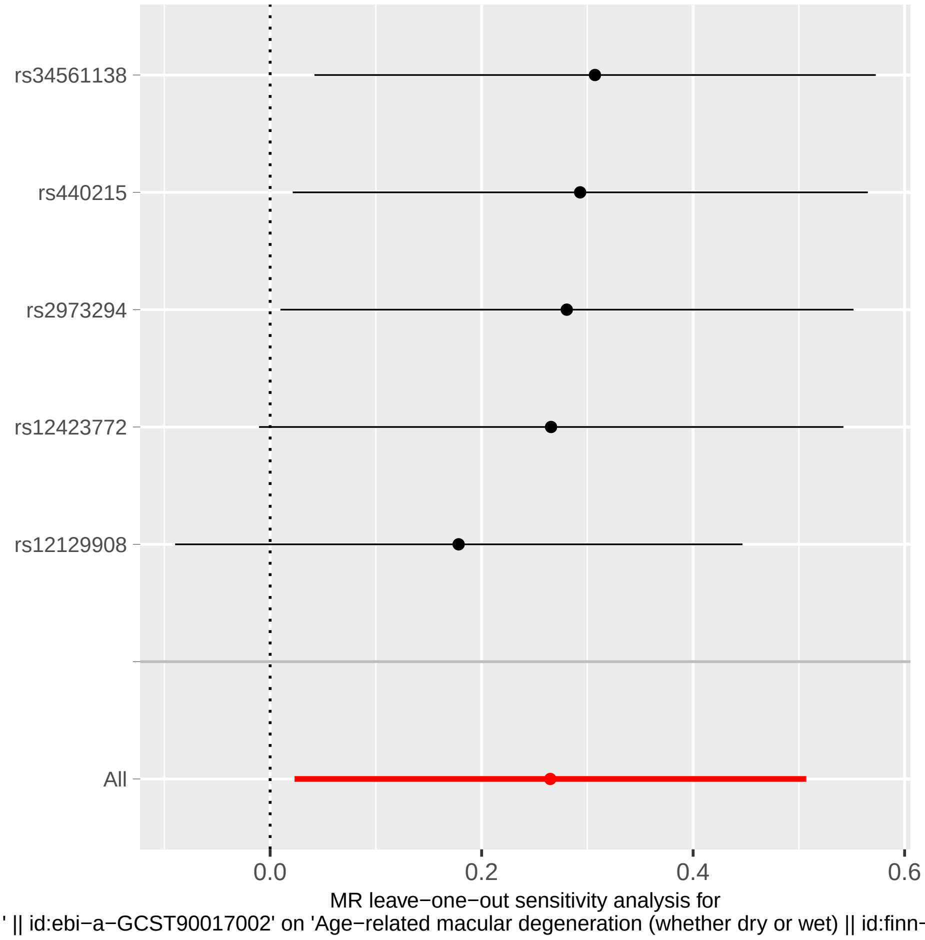

rs34561138
rs440215
rs2973294
rs12423772
rs12129908
All
0.0
0.2
0.4
0.6
MR leave−one−out sensitivity analysis for
' || id:ebi−a−GCST90017002' on 'Age−related macular degeneration (whether dry or wet) || id:finn−

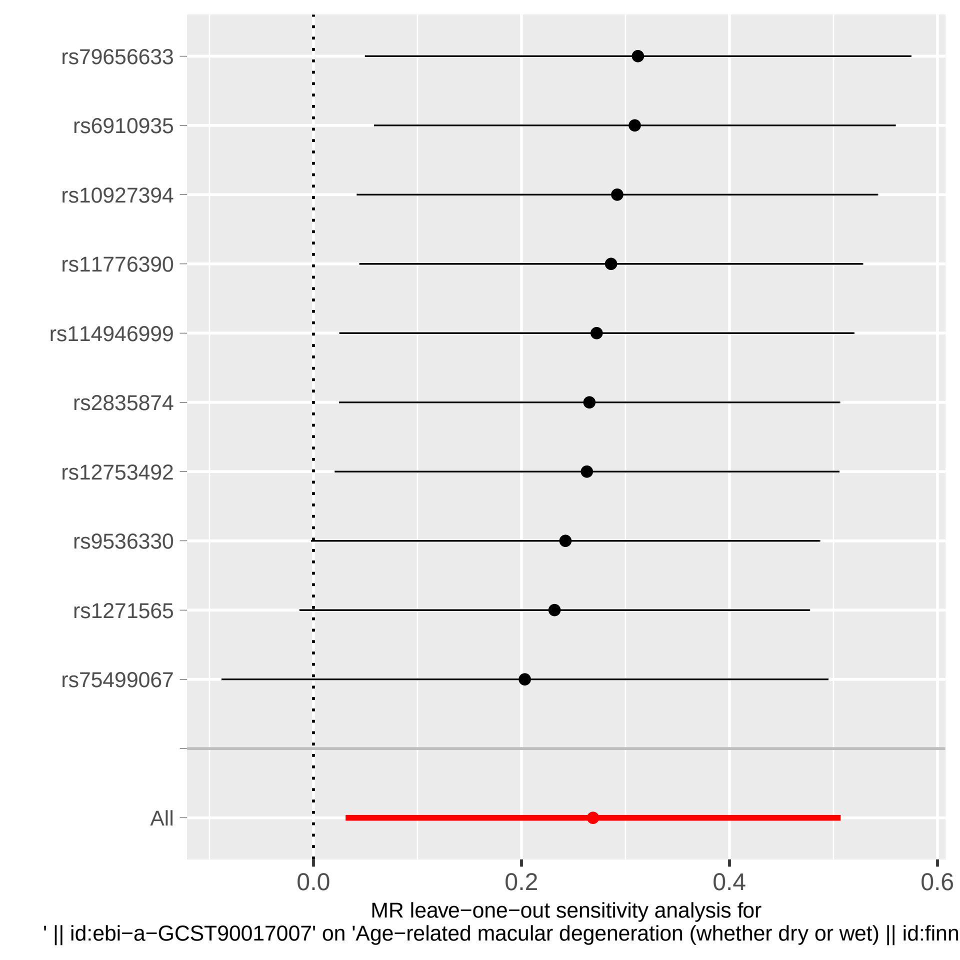
rs79656633
rs6910935
rs10927394
rs11776390
rs114946999
rs2835874
rs12753492
rs9536330
rs1271565
rs75499067
All
0.0
0.2
0.4
0.6
MR leave−one−out sensitivity analysis for
' || id:ebi−a−GCST90017007' on 'Age−related macular degeneration (whether dry or wet) || id:finn

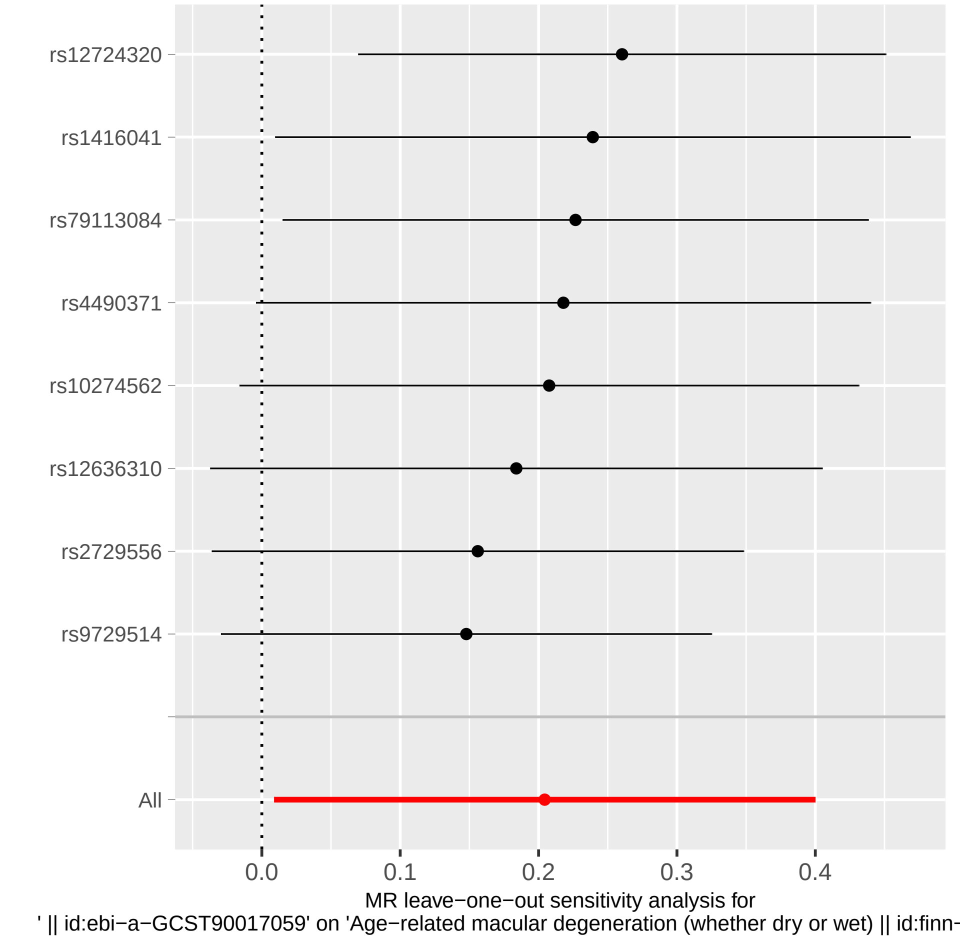
rs12724320
rs1416041
rs79113084
rs4490371
rs10274562
rs12636310
rs2729556
rs9729514
All
0.0
0.1
0.2
0.3
0.4
MR leave−one−out sensitivity analysis for
' || id:ebi−a−GCST90017059' on 'Age−related macular degeneration (whether dry or wet) || id:finn−

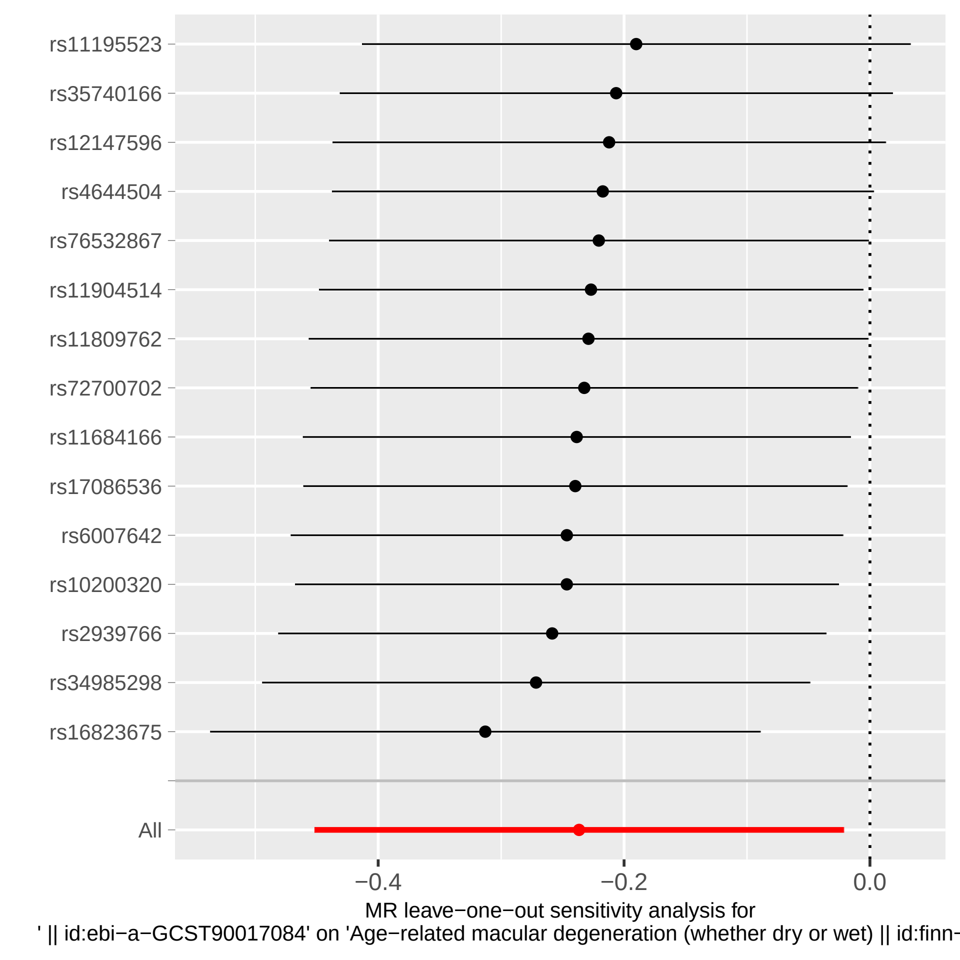
rs11195523
rs35740166
rs12147596
rs4644504
rs76532867
rs11904514
rs11809762
rs72700702
rs11684166
rs17086536
rs6007642
rs10200320
rs2939766
rs34985298
rs16823675
All
−0.4
−0.2
0.0
MR leave−one−out sensitivity analysis for
' || id:ebi−a−GCST90017084' on 'Age−related macular degeneration (whether dry or wet) || id:finn−
